# Molecular Mechanism of SR Protein Kinase 1 Inhibition by the Herpes Virus Protein ICP27

**DOI:** 10.1128/mBio.02551-19

**Published:** 2019-10-22

**Authors:** Richard B. Tunnicliffe, William K. Hu, Michele Y. Wu, Colin Levy, A. Paul Mould, Edward A. McKenzie, Rozanne M. Sandri-Goldin, Alexander P. Golovanov

**Affiliations:** a Manchester Institute of Biotechnology and Department of Chemistry, Faculty of Science and Engineering, The University of Manchester, Manchester, United Kingdom; b Department of Microbiology and Molecular Genetics, School of Medicine, University of California, Irvine, California, USA; c Biomolecular Analysis Core Facility, Faculty of Biology, Medicine and Health, University of Manchester, United Kingdom; University of North Carolina, Chapel Hill

**Keywords:** phosphorylation, splicing, herpes simplex virus, structure, phosphorylation, SR protein kinase

## Abstract

Serine-arginine (SR) protein kinase 1 (SRPK1) catalyzes the phosphorylation of SR proteins, which are a conserved family of splicing factors that contain a domain rich in arginine and serine repeats. SR proteins play important roles in constitutive pre-mRNA splicing and are also important regulators of alternative splicing. During herpes simplex virus infection, SRPK1 is inactivated and its cellular distribution is markedly altered by interaction with the viral protein ICP27, resulting in hypophosphorylation of SR proteins. Mutational analysis previously showed that the RGG box motif of ICP27 is required for interaction with SRPK1; however, the mechanism for the inhibition and the exact role of the RGG box was unknown. Here, we used solution nuclear magnetic resonance (NMR) spectroscopy and isothermal titration calorimetry (ITC) to demonstrate that the isolated peptide comprising the RGG box of ICP27 binds to SRPK1 with high affinity, competing with a native substrate, the SR repeat region of SR protein SRSF1. We determined the crystal structure of the complex between SRPK1 and an RGG box peptide, which revealed that the viral peptide binds to the substrate docking groove, mimicking the interactions of SR repeats. Site-directed mutagenesis within the RGG box further confirmed the importance of selected arginine residues for interaction, relocalization, and inhibition of SRPK1 *in vivo*. Together these data reveal the molecular mechanism of the competitive inhibition of cellular SRPK1 by viral ICP27, which modulates SRPK1 activity.

## Abstract (precis)

Serine arginine (SR) proteins are a family of mRNA regulatory proteins that can modulate spliceosome association with different splice sites and therefore regulate alternative splicing. Phosphorylation within SR proteins is necessary for splice-site recognition, and this is catalyzed by SR protein kinase 1 (SRPK1). The herpes simplex virus (HSV-1) protein ICP27 has been shown previously to interact with and downregulate SRPK1 activity *in vivo*; however, the molecular mechanism for this interaction and inhibition was unknown. Here, we demonstrate that the isolated peptide fragment of ICP27 containing RGG box binds to SRPK1 with high affinity, and competes with a native cellular substrate. Elucidation of the SRPK1-RGG box crystal structure further showed that a short palindromic RGRRRGR sequence binds in the substrate docking groove of SRPK1, mimicking the binding of SR repeats of substrates. These data reveal how the viral protein ICP27 inactivates SRPK1, promoting hypophosphorylation of proteins regulating splicing.

## INTRODUCTION

Alternative pre-mRNA splicing is a fundamental mechanism that increases the diversity of protein isoforms expressed by eukaryotes, and defects in its regulation are associated with multiple disorders, including cancer (1, 2). Serine-arginine (SR) splicing factors modulate spliceosome association with different splice sites and therefore regulate alternative splicing (3). One of the key regulators of SR protein activity in humans is serine-arginine protein kinase 1 (SRPK1), which phosphorylates SR repeat sequences of SR proteins promoting both their localization in the nucleus and splicing activity (4–6). Often more than one string of SR repeats are clustered together separated by short non-SR sequences in regions termed RS domains. The structure of SRPK1 is composed of an N-terminal domain containing the active site linked to a C-terminal domain that forms a docking groove, which recognizes SR repeats (7, 8). Substrates such as the prototype SR protein SRSF1 (ASF/SF2) bind tightly to the SRPK1 docking groove, with a dissociation constant of the order of 50 nM (9–11). SRPK1 can phosphorylate multiple SR repeats, using a processive mechanism whereby the substrate peptide slides through the docking groove toward the ATP binding pocket in a C- to N-terminal direction (4, 12–14). Alternatively, SRPK1 can also use a distributive mechanism, in which the enzyme recognizes short SR sequences, phosphorylates them, and dissociates from a substrate (4). The distributive mechanism has been characterized in the SR protein Tra2β, which contains only a tetra-SR repeat and isolated SR dipeptides, and mutagenesis experiments indicated that the SRPK1 docking groove was dispensable for this mode of catalysis (15).

SRPK1 also acts with other cellular SR protein kinases, which provide an additional layer of regulation by tuning substrate regiospecific phosphorylation and nuclear-cytoplasmic distribution. For example, the octa-SR repeat containing the N-terminal half of the RS domain of SRSF1 is phosphorylated by SRPK1, whereas a different kinase from the CLK family acts on the C-terminal half (16, 17). SRPK1 does not contain a nuclear localization sequence (NLS), so it is predominantly cytoplasmic and SR phosphorylation facilitates SR protein nuclear import via a specific transportin protein (18–20). Subsequently CLKs (which have an NLS) phosphorylate SR proteins in the nucleus. Additionally CLKs can also bind SRPK1 and facilitate the latter’s passage into the nucleus, where the interaction of SRPK1 with CLKs facilitates the release of SR proteins from CLK1 (21). Therefore, the ternary mixture of SRPK1, CLKs, and SR proteins affects the latter’s cellular distribution and regiospecific phosphorylation, which modulates recognition of splice sites and in turn recruits spliceosome proteins thus mediating alternative splicing (21, 22). SRPK1 action on SR proteins has also been shown to facilitate angiogenesis and neovascularization by promoting the production of proangiogenic isoforms of the vascular endothelial growth factor (VEGF) and therefore was recognized as a promising drug target for a variety of diseases associated with deregulated alternative splicing (1, 23). SRPK1 also acts as a general signal modulator besides its role in regulated splicing. Another regulatory role of SRPK1 is its interaction with Akt kinases, which are active as phosphoproteins, and also PHLPP1, a phosphatase that inactivates Akt (24). Aberrant SRPK1 expression can lead to cancer progression. SRPK1 underexpression leads to Akt hyperactivation due to a lack of the downregulatory ternary complex formation with PHLPP1. Conversely, overexpression of SRPK1 can shift the equilibrium toward formation of the SRPK1-PHLPP1 heterodimer, which becomes dominant over the regulatory ternary complex, thus also disabling control of Akt, and leading to enhanced anchorage-independent cell growth (24).

**FIG 1:**
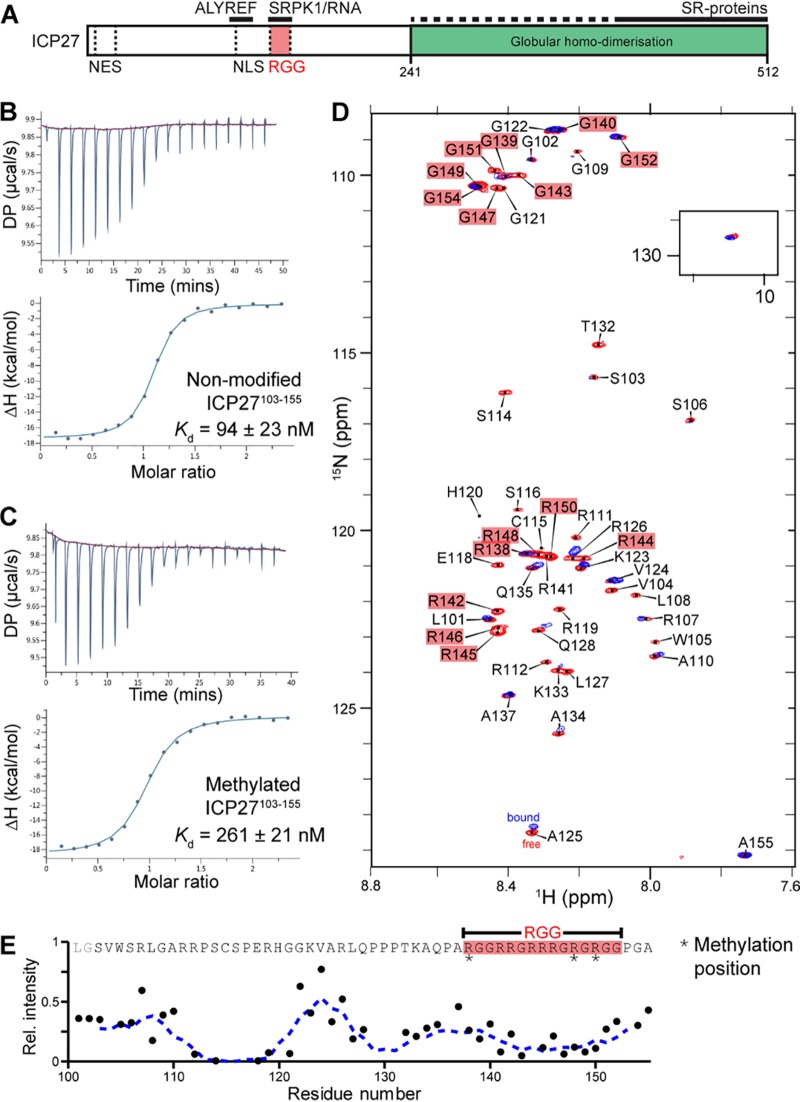
SRPK1 binds to the RGG box of ICP27 *in vitro*. (A) Schematic of subdomains and interaction sites within HSV-1 ICP27. (B) Affinity quantification of SRPK1 interaction with unmodified ICP27^103–155^. (C) As in panel B but with methylated ICP27^103–155^. (D) ^1^H-^15^N correlation spectra of [^15^N]ICP27^103–155^ in the absence (red) and presence (blue) of a stoichiometric equivalent of SRPK1. (E) Binding site mapped by analysis of relative signal intensity change upon binding, *I^B^*/*I^F^*. The decrease in signal intensity of a protein occurs upon interaction with SPRK1.

A number of viruses, such as herpes simplex virus 1 (HSV-1), hepatitis B virus (HBV), and human papillomavirus (HPV) express proteins that bind to SRPK1, and some of these interacting proteins can also modulate kinase activity, which shifts host mRNA processing in favor of viral transcripts (25–27). HSV-1 protein ICP27 was the first viral protein identified as an SRPK1 regulator. It induces hypophosphorylation of SR proteins and redistribution of the kinase into the nucleus (27). To facilitate viral replication, ICP27 plays a central role by interacting with and hijacking cellular proteins that function throughout the RNA maturation process (28). Most HSV-1 genes lack introns and are inefficiently expressed without the action of ICP27, which promotes their expression in favor of intron-containing cellular transcripts (29–33). HSV-1 ICP27 and its HSV-2 homolog can trigger alternative splicing of specific transcripts (34–37). ICP27 contains a C-terminal region called the ICP27 homology domain, which is a conserved globular domain that mediates homo-oligomerization (38–42) (Fig. 1A), which interacts with cellular proteins that function in RNA transcription and export, including SR proteins (27, 28, 43). The N-terminal domain of ICP27 is an intrinsically disordered region (IDR), which contains short binding motifs such as for the TREX protein ALYREF (residues 103 to 112) (30, 44) and an RGG box (residues 138 to 152) rich in arginines and glycines that was shown to be necessary for RNA and SRPK1 interactions (27, 45–48). Posttranslational modifications within the RGG box carried out by PRMT1 (protein arginine methyltransferase 1), which result in methylation of R138, R148, and R150 (48–50), modulate SRPK1 interactions (51); however, the details of the ICP27-SRPK1 interaction and therefore the reason for this modulation were not identified. The N-terminal IDR of ICP27 also contains both nuclear export and nuclear localization sequences that facilitate nuclear-cytoplasmic shuttling, particularly in later stages of HSV-1 infection (33, 47, 52, 53). In uninfected cells, SRPK1 is predominately cytoplasmic (54); however, HSV-1 infection and the subsequent interaction of ICP27 with SRPK1 shift the latter into the nucleus, resulting in SR protein hypophosphorylation and disrupted spliceosome assembly, thus indicating SRPK1 could be inhibited by ICP27 (27). As the molecular details of the ICP27-SRPK1 complex and the mechanism of kinase modulation and the precise role of the RGG box were unknown, we have characterized its structure and explored the interaction via biophysical and *in vivo* techniques to gain insights into the mode of action of the viral protein.

## RESULTS

### Mapping and quantification of SRPK1 binding to ICP27 peptide fragments containing the RGG box motif.

The RGG box in full-length ICP27 (Fig. 1A) was previously identified by mutagenesis experiments as the segment necessary for interaction with SRPK1 (27), with the binding modulated by arginine methylation (51). To determine if the isolated RGG box motif can bind SRPK1, we obtained a synthetic peptide of ICP27^103–155^, which comprises the RGG box plus the adjacent ALYREF-binding and NLS regions (Fig. 1A), and purified full-length SRPK1 tagged with an N-terminal GB1 solubility tag (GB1-SRPK1). Their interaction was measured by isothermal titration calorimetry (ITC), which indicated a *K_d_* (dissociation constant) of 94 ± 23 nM (Fig. 1B; see Table S1 in the supplemental material). The experiment was also performed with a synthetic peptide methylated within the RGG box at arginine positions 138, 148 and 150, which weakened the interaction 3-fold with a *K*_d_ of 262 ± 21 nM (Fig. 1C; see Table S2 in the supplemental material). To further confirm and map the RGG box interaction with SRPK1, we expressed and purified ^15^N-labeled ICP27^103–155^ and using the established backbone assignment (55) carried out nuclear magnetic resonance (NMR) signal perturbation mapping by comparison of ^1^H-^15^N correlation spectra in the presence and absence of unlabeled GB1-SRPK1 (Fig. 1E). The data showed substantial signal broadening, typical of somewhat dynamic binding of parts of the polypeptide chain to a slowly tumbling high-molecular-weight species (Fig. 1D), particularly within ICP27 residues 112 to 121 and 138 to 152, the latter comprising the RGG box. These solution data together confirmed that SRPK1 binds with high affinity to a relatively short region of ICP27 containing the RGG box, with possible further contributions from nearby residues 112 to 121, whereas other residues retain significant flexibility when the peptide is bound. The ITC measurement of 94 ± 23 nM is comparable to 50 ± 25 nM reported previously for RS domain binding of a native substrate, SRSF1, determined using *in vitro* phosphorylation assays (9–11).

10.1128/mBio.02551-19.6 TABLE S1 The thermodynamic parameters as measured by isothermal titration calorimetry (ITC) of the interaction between SRPK1 and nonmethylated ICP27^103–155^ peptide are shown. Download Table S1, DOCX file, 0.1 MB. Copyright © 2019 Tunnicliffe et al. 2019 Tunnicliffe et al. This content is distributed under the terms of the Creative Commons Attribution 4.0 International license.

10.1128/mBio.02551-19.7 TABLE S2 The thermodynamic parameters as measured by isothermal titration calorimetry of the interaction between SRPK1 and methylated ICP27^103–155^ peptide are shown. Download Table S2, DOCX file, 0.1 MB. Copyright © 2019 Tunnicliffe et al. 2019 Tunnicliffe et al. This content is distributed under the terms of the Creative Commons Attribution 4.0 International license.

### NMR directly confirms competition between SRSF1 and ICP27^103–155^ for SRPK1 binding.

**FIG 2:**
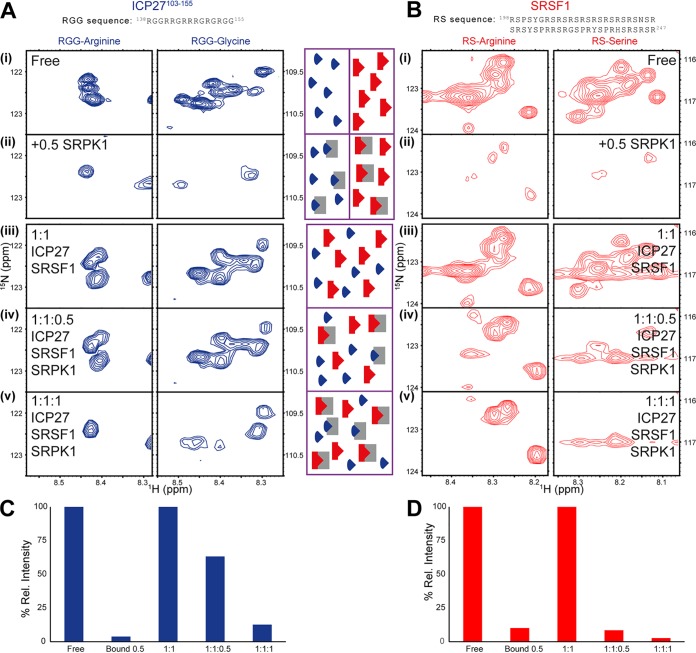
IDIS-NMR experiments (56) indicate competition for SRPK1 binding between SRSF1 and the RGG box region of ICP27. Reporter signals for SRPK1 binding are shown for (A) RGG box within [^13^C, ^15^N]ICP27^103–155^ and (B) RS region from full-length [^15^N]SRSF1. (i) ICP27 or SRSF1 in isolation, (ii) ICP27 or SRSF1 with half a stoichiometric equivalent of unlabeled GB1-SRPK1 added, (iii) 1:1 mixture of ICP27 and SRSF1, (iv) 1:1:0.5 mixture of ICP27, SRSF1 and GB1-SRPK1, (v) 1:1:1 mixture of ICP27, SRSF1, and GB1-SRPK1. The normalized relative intensities of reporter signals *I*^*rel*^ measured from the spectra shown in panels A and B are plotted in panel C for ICP27 and panel D for SRSF1. The decrease in signal intensity of a protein occurs upon interaction with SPRK1, and comparison of signals from ICP27 and SFSR1 within the same sample tube therefore provides an indication of binding preference. The central scheme illustrates the binding scenarios for each sample, with GB1-SRPK1, ICP27^103–155^, and SRSF1 molecules colored gray, blue, and red, respectively.

Both the viral RGG box and RS domain found in the native cellular substrate SRSF1 bear some superficial sequence similarity (i.e., both are arginine-rich), although only the RS domain is a substrate for the SRPK1 kinase and becomes phosphorylated (5, 6). Our measurements using ITC and the data from the literature (9–11) do suggest that the binding of the RGG box regions of ICP27 and the RS domain of SRSF1 with SRPK1 may be comparable, but which of them is stronger, and whether they are competitive with each other, needed to be established under identical experimental conditions. To explore if these two proteins bind SRPK1 competitively, we used a direct approach, in which all three proteins, in differentially labeled forms, were mixed together in stoichiometric amounts, and isotopically discriminated (IDIS)-NMR (56) was used to detect which of the protein complexes is formed preferentially. Initial control ^1^H-^15^N heteronuclear single quantum coherence (HSQC) and transverse relaxation-optimized spectroscopy (TROSY) experiments established fingerprint spectra of uniformly ^15^N-labeled full-length SRSF1, acquired in the presence and absence of unlabeled GB1-SRPK1. These characteristic spectral patterns enabled us to recognize the presence of SRSF1-SRPK1 complex in solution. The SRSF1 spectrum was dominated by sharp poorly dispersed amide signals typical of IDRs, especially those from the SR repeats, while lower-intensity dispersed amide signals could be observed in TROSY spectra typical of folded globular regions (see Fig. S1 in the supplemental material). Upon addition of GB1-SRPK1, the SR repeat signals were broadened, substantially weakening their signal intensity along with several other peaks from the IDRs (Fig. 2). The ICP27^103–155^ mapping experiment described earlier acted as a reference for the signal pattern, which was characteristic for SRPK1 binding to the RGG box (Fig. 1): observing similar characteristic signal patterns in multicomponent protein mixtures enables us to infer the presence of the SRPK1-RGG box complex. For the following IDIS-NMR experiments, a stoichiometric mixture of differentially labeled 50 μM [^13^C,^15^N]ICP27^103–155^ and 50 μM [^15^N]SRSF1 was first prepared, and spectra were recorded, which showed only minor signal perturbations compared to the proteins in isolation, indicating that residues 103 to 155 of ICP27 do not interact with SRSF1 (Fig. 2 [full spectra in Fig. S2 and Fig. S3 in the supplemental material]). Addition of a substoichiometric amount (25 μM) of nonlabeled GB1-SRPK1 resulted in strong signal perturbations in SRSF1 SR repeat signals, whereas in ICP27, the RGG box signals were relatively unperturbed, indicative of preferential binding of SRPK1 to the RS domain of SRSF1. This was also evident by comparison of the signal intensities measured relative to the nonbound proteins at the middle point of this titration, where SRSF1, ICP27, and SRPK1 were mixed in a stoichiometric ratio of 1:1:0.5, as the SRSF1 signal is clearly much weaker than ICP27 (Fig. 2C and D). Addition of more GB1-SRPK1 up to 50 μM final concentration produced a stoichiometric ternary mixture (1:1:1) of SRPK1, SRSF1, and ICP27^103–155^, resulting in some further SRSF1 SR signal broadening while significantly inducing substantial ICP27 RGG box broadening. The further changes observed particularly were suggestive of SRPK1 binding to the RS domain approaching saturation alongside significant binding to the RGG box. Together the data indicate that although SRPK1 has a marginal preference for the SR regions of full-length SRSF1 over a short ICP27^103–155^ peptide, the latter is still able to compete for SRPK1 binding (Fig. 2C and D). This experiment, in which all three proteins are present at equal concentration in solution and are allowed to compete for binding with each other, demonstrates the presence of this competition directly.

10.1128/mBio.02551-19.2 FIG S1 TROSY ^1^H-^15^N-NMR correlation spectrum of uniformly ^15^N-labeled full-length SRSF1. The same spectrum is shown with contour levels adjusted to illustrate differential peak heights. (A) The high-contour level only reveals sharp poorly dispersed signals from intrinsically disordered termini, including the RS repeats labeled with black dashed boxes. (B) Low-contour view where broad and dispersed signals from globular RRM domains can be observed in addition to sharp signals. Download FIG S1, DOCX file, 0.2 MB. Copyright © 2019 Tunnicliffe et al. 2019 Tunnicliffe et al. This content is distributed under the terms of the Creative Commons Attribution 4.0 International license.

10.1128/mBio.02551-19.3 FIG S2 Interaction of uniformly ^15^N-labeled SRSF1with unlabeled SRPK1 monitored by HSQC and IDIS-NMR ^1^H-^15^N correlation spectra. Dashed boxes mark positions of overlapped signals from the RS repeat regions used in intensity analysis. (A) Superposition of HSQC of SRSF1 in the presence (blue) and absence (red) of SRPK1, the kinase at half the concentration of SRSF1. (B) IDIS-HSQC ^15^N subspectra of 1:1 [^15^N]SRSF1 and [^15^N, ^13^C]ICP27^103–155^. (C) The same sample as panel B with the addition of SRPK1 to a 0.5 stoichiometric equivalent. (D) The same sample as panel C with a further addition of SRPK1 to equimolar concentration giving a ternary 1:1:1 mixture of unlabeled SRPK1, [^15^N]SRSF1, and [^15^N, ^13^C]ICP27^103–155^. Download FIG S2, DOCX file, 0.1 MB. Copyright © 2019 Tunnicliffe et al. 2019 Tunnicliffe et al. This content is distributed under the terms of the Creative Commons Attribution 4.0 International license.

10.1128/mBio.02551-19.4 FIG S3 Interaction of uniformly ^13^C, ^15^N-labeled ICP27^103–155^ with unlabeled SRPK1 monitored by HSQC and IDIS-NMR ^1^H-^15^N correlation spectra. Dashed boxes mark positions of overlapped signals from the RGG box signals used in intensity analysis. (A) Superposition of HSQC of ICP27^103–155^ in the presence (blue) and absence (red) of SRPK1, with the kinase at half the concentration of ICP27^103–155^. (B) IDIS-HSQC ^13^C, ^15^N subspectra of 1:1 [^15^N]SRSF1 and [^15^N, ^13^C]ICP27^103–155^. (C) Same sample as panel B with addition of SRPK1 to a 0.5 stoichiometric equivalent. (D) Same sample as panel C with a further addition of SRPK1 to an equimolar concentration giving a ternary 1:1:1 mixture of unlabeled SRPK1, [^15^N]SRSF1, and [^15^N, ^13^C]ICP27^103–155^. Download FIG S3, DOCX file, 0.2 MB. Copyright © 2019 Tunnicliffe et al. 2019 Tunnicliffe et al. This content is distributed under the terms of the Creative Commons Attribution 4.0 International license.

### The crystal structure of ICP27 RGG box peptide bound to the docking groove of SRPK1.

Having demonstrated that the peptide encompassing the ICP27 RGG box motif can directly bind to SRPK1 and is sufficient for high-affinity binding, we determined the structure of the complex by X-ray crystallography. The SRPK1 ΔNS1 protein construct, which was used previously for crystallization studies (7, 8, 57), was purified and incubated with ICP27^137–152^ RGG box peptides. Separate crystal trials using either unmodified or methylated peptides were performed, but cocrystallization was only successful with the unmodified ICP27^137–152^ peptide, which diffracted to 2.8 Å (see Table S3 in the supplemental material). Coordinates were deposited in the Protein Data Bank, under accession code 6FAD.

10.1128/mBio.02551-19.8 TABLE S3 Data were collected from single cryo-frozen crystal SRPK1 ΔNS1-RGG box complex at beamline i04-1 and were indexed, scaled, and integrated with Xia2. The X-ray crystallography data collection and refinement statistics for the SRPK1-ICP27^137–152^ structure are shown. Download Table S3, DOCX file, 0.1 MB. Copyright © 2019 Tunnicliffe et al. 2019 Tunnicliffe et al. This content is distributed under the terms of the Creative Commons Attribution 4.0 International license.

**FIG 3:**
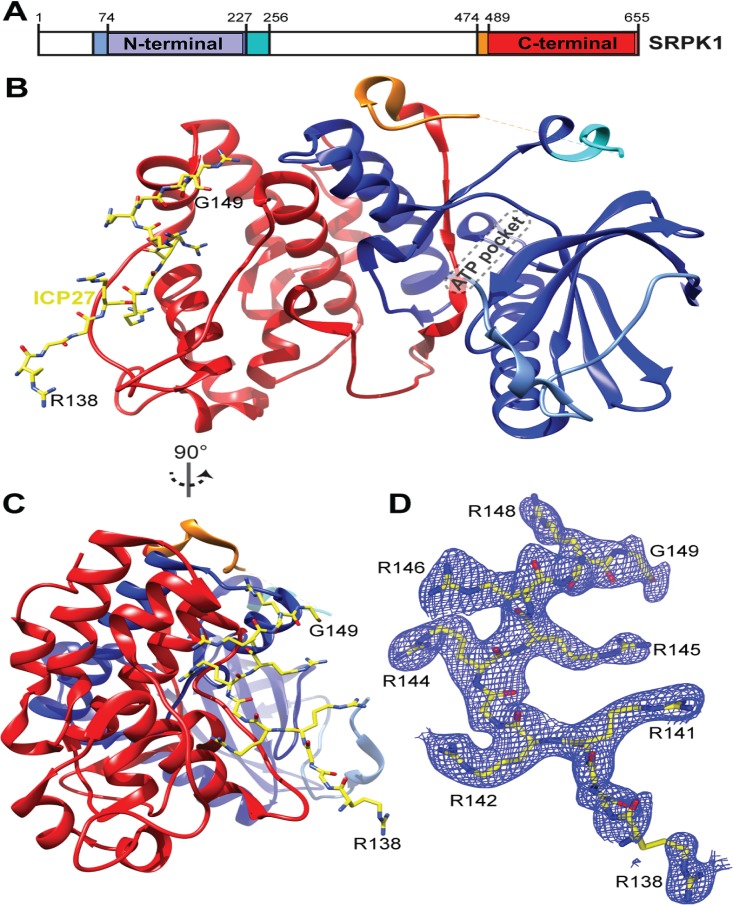
Structure of SRPK1 with RGG box peptide ICP27^137–152^ bound in the substrate docking groove. (A) Schematic of SRPK1 domains. (B) Crystal structure of the SRPK1 in complex with ICP27^137–152^. SRPK1 is colored as panel A, ICP27 is yellow. (C) As in panel A but view rotated 90°. (D) 2*F*_o_ − *F*_c_ electron density map of RGG peptide colored blue, contoured to 1σ orientated as in panel B.

**FIG 4:**
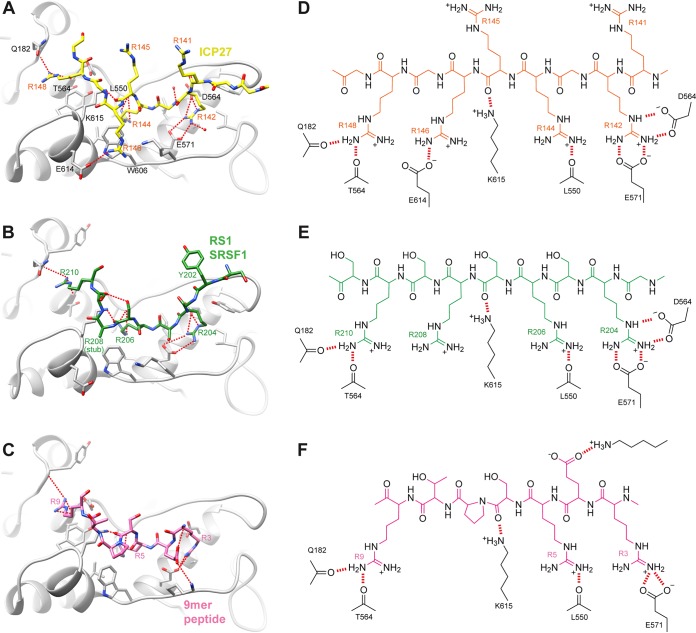
Comparison of SRPK1 docking groove interactions. (A) Detail of the RGG box peptide bound within the SRPK1 docking groove, plus comparative views of SRPK1 substrate complexes: (B) SRSF1 RS1 region (8) or (C) 9-mer docking peptide of membrane-associated guanylate kinase (7). SRPK1 is shaded gray, and bound peptides are colored. Intermolecular hydrogen bonds or salt bridges are represented by red dashes. Chemical schematics of the interactions shown in panels A to C are shown in panels D to F, respectively.

The asymmetric unit (ASU) contained four biological assemblies composed of SRPK1 bound to an RGG box peptide. Residues 142 to 148 of ICP27, a 7-mer palindrome, RGRRRGR, form the majority of the intermolecular contacts with the docking groove of SRPK1 (Fig. 3), and were observed in all 4 copies of the biological assembly within the ASU with the same binding mode and residue register. One out of the four copies of RGG box peptides (chain E) forms additional crystal contacts, in which residues 138 to 141 bind SRPK1 chain D within an adjacent ASU, while residues 142 to 149 form “typical” contacts with SRPK1 chain A within the same ASU (see Fig. S4 in the supplemental material), as observed in other copies of the assembly. The complex formed by chains A and E of SRPK1 and ICP27, respectively, was the most complete and will be referred to in further discussions (see Table S4 in the supplemental material). Within SRPK1, four arginine side-chain binding sites were identified, which contained R142, R144, R146, and R148 of ICP27 (Fig. 4D): the R142 side chain forms partially buried ionic contacts with E564 and E571, the R144 side chain is again partially buried and forms a hydrogen bond with the backbone carbonyl of L550, R146 forms a stacking interaction with the side chain of W606, and finally, R148 forms hydrogen bonds with backbone carbonyls of Q182 and T546. Comparison of the SRPK1-RGG box complex (Fig. 4D) with other structures of SRPK1 in complex with SRSF1-RRM2 and RS1 domain (PDB code 3BEG) (Fig. 4E) and membrane-associated guanylate kinase 9-mer docking peptide (PDB code 1WBP) (Fig. 4F) indicated that the recognition of the ICP27 peptide closely resembles these other docking groove complexes. In particular, the arginine residues from the RS domain of SRSF1 and ICP27 residues R142, R144, R146, and R148 form near-identical polar interactions and salt bridges with SRPK1 (Fig. 4). While the C terminus of the peptide turns and points toward the active site, it is still positioned quite far away from the active site, not blocking it directly (Fig. 3B). Specifically, the Cα of G149, which is the C-terminal residue observed in the electron density of the RGG box, is located approximately 30 Å from the ATP binding pocket of SRPK1. The similarities in how the docking groove of SRPK1 recognizes stretches of alternating arginines present both within the native RS domain substrate and the viral RGG box are striking, and they corroborate the competitive nature of ICP27 and SRSF1 interactions with SRPK1 observed by IDIS-NMR experiments in solution.

10.1128/mBio.02551-19.5 FIG S4 One ICP27 RGG box peptide out of four present in the asymmetric unit (ASU) forms additional crystal packing contacts. Chains A (blue ribbons) and E (green sticks) are within the same ASU and chain D (pink ribbons) in an adjacent ASU. Chain E bridges the two SRPK1 molecules, with ICP27 N-terminal residues 138 to 141 forming apparent nonnative contacts. A 2F_o_ − F_c_ electron density map for the ICP27 peptide is shown as blue chicken wire scaled to 1σ. (A) Overview showing the complete chains of SRPK1 chains A and D plus ICP27 chain E. (B) Zoomed-in view with residues labeled that make crystal packing contact in chains E and D. Download FIG S4, DOCX file, 2.6 MB. Copyright © 2019 Tunnicliffe et al. 2019 Tunnicliffe et al. This content is distributed under the terms of the Creative Commons Attribution 4.0 International license.

10.1128/mBio.02551-19.9 TABLE S4 The composition of the four molecular assemblies that comprise the asymmetric unit in the X-ray structure of ICP27^137–152^ in complex with SRPK1 ΔNS1 are listed. Download Table S4, DOCX file, 0.1 MB. Copyright © 2019 Tunnicliffe et al. 2019 Tunnicliffe et al. This content is distributed under the terms of the Creative Commons Attribution 4.0 International license.

### ICP27 RGG box mutants do not interact with or change cellular localization of SRPK1 *in vivo*.

**FIG 5:**
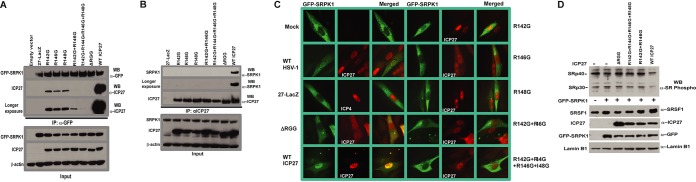
ICP27 RGG box mutants do not interact with SRPK1 and do not inhibit its activity *in vivo*. HeLa cells cotransfected with GFP-SRPK1 and WT ICP27 or RGG box mutants were infected for 8 h with 27-LacZ. Immunoprecipitation was performed using anti-GFP antibody. Western blots were probed with anti-GFP or anti-ICP27 antibody and anti-β-actin as a loading control. (B) HeLa cells transfected with WT and ICP27 RGG box mutants were infected with 27-LacZ for 8 h. Immunoprecipitation was performed with anti-ICP27 antibody. Western blots were probed with anti-SRPK1 or anti-ICP27 antibody as indicated and anti-β-actin as a loading control. (C) RSF cells were transfected with GFP-SRPK1 and 16 h later were mock infected, infected with WT HSV-1 or 27-LacZ, or were cotransfected with GFP-SRPK1 and WT ICP27 or ICP27 RGG box mutants and infected 16 later with 27-LacZ. Cells were fixed 6 h later and immunofluorescent staining was performed with anti-ICP27. ICP4 antibody was used to stain the 27-LacZ sample to mark the nucleus. GFP fluorescence was visualized directly. The panels shown are representative of ∼80% of cells visualized. (D) HeLa cells were transfected with GFP-SRPK1 and WT ICP27 or RGG box mutants and were infected with 27-LacZ 16 h after transfection for 8 h. Portions of cell lysates were fractionated by SDS-PAGE, and Western blots were probed with antiphosphoepitope SR antibody, anti-SF2 (SRSF1), anti-ICP27 antibody, and anti-lamin B1 antibody as a loading control.

ICP27 mutants were constructed to target sites within the RGG box that contact SRPK1 in the crystal structure. Arginines at positions 142, 144, 146, and 148 were changed to glycine (G) as single (R142G, R146G, or R148G), double (R142G R146G), and quadruple (R142G R144G R146G R148G) substitutions. The ICP27 mutant plasmids were cotransfected with a plasmid expressing green fluorescent protein (GFP)-SRPK1 and subsequently infected with ICP27 null mutant virus 27-LacZ to recapitulate the conditions of infection. At 8 h after infection, lysates were immunoprecipitated with anti-GFP antibody, and samples were analyzed by Western blotting and probed with antibody to ICP27 (Fig. 5A). Wild-type (WT) ICP27 was efficiently coimmunoprecipitated with GFP-SRPK1; however, this was substantially reduced for ICP27 R142G, R146G, and R148G single mutants and the R142G R146G double mutant, whereas the R142G R144G R146G R148G quadruple mutant was not detected even after a longer exposure, as was the case for the ΔRGG mutant in which the RGG box was deleted (Fig. 5A). ICP27 interaction with endogenous SRPK1 was also analyzed. Cells were transfected with WT and ICP27 mutant plasmids and infected with 27-LacZ as described above. Immunoprecipitation was performed with anti-ICP27 antibody and the blots were probed with antibody to SRPK1 (Fig. 5B). SRPK1 was only coimmunoprecipitated with WT ICP27 and was not detected with any of the mutants.

Although SRPK1 is predominantly located in the cytoplasm, where it phosphorylates SR proteins so that they are transported to the nucleus by an SR-specific transportin protein, a smaller amount of SRPK1 is also located in the nucleus (21). Also, under some conditions, SRPK1 nuclear levels become increased. For example, epidermal growth factor signaling increases nuclear levels of SRPK1 through the Akt-dependent release of chaperones from the spacer insert domain of SRPK1, which appears to restrict SRPK1 to the cytoplasm (58). It was previously shown that during HSV-1 infection ICP27 relocalizes SRPK1 from the cytoplasm to the nucleus and the RGG box is required for this relocalization (27, 51). To determine if the ICP27 RGG box mutants were able to relocalize SRPK1, cells were cotransfected with GFP-SRPK1 and WT or ICP27 mutant plasmids and were subsequently infected with 27-LacZ, and 6 h later, cells were fixed and stained for immunofluorescence analysis. In mock-infected cells and cells infected with 27-LacZ, SRPK1 was predominantly cytoplasmic, whereas in WT HSV-1 KOS infected cells, SRPK1 was also clearly localized in the nucleus (Fig. 5C). In cells transfected with WT ICP27 plasmid and infected with 27-LacZ, SRPK1 was seen to be prominently nuclear. In contrast, SRPK1 was largely cytoplasmic in cells transfected with the R142G, R146G, R142G R146G, and R142G R144G R146G R148G mutants, similar to what was observed for ΔRGG-, mock-, and 27-LacZ-infected cells (Fig. 5C). As low levels of SRPK1 are usually present in the nucleus of uninfected cells, some weak colocalization can be observed—for example, with R142G; however, it is clear that SRPK1 was not efficiently recruited to the nucleus by ICP27 RGG box mutants. Similar results were seen at 4 and 8 h after infection (data not shown).

We showed previously that ICP27 inhibited SRPK1 phosphorylation of SR proteins via *in vitro* SRPK1 kinase assays, and the interaction of ICP27 and SRPK1 resulted in hypophosphorylation of SR proteins *in vivo* (27). To determine how the ICP27 RGG box mutants would affect SR protein phosphorylation *in vivo*, cells were cotransfected with GFP-SRPK1 and WT ICP27 or ΔRGG, R142G R144G R146G R148G, and R142G R146G mutants and were subsequently infected with 27-LacZ (Fig. 5D). Western blots were probed with an anti-phosphoepitope SR protein antibody that recognizes phosphorylated serines within SR repeats. The level of phosphorylation for SRp40 (SRSF5) was reduced in cells transfected with WT ICP27, but not in those transfected with the mutants (Fig. 5D). Similarly, phosphorylation of SRp30 (SRSF1) was reduced with WT ICP27, indicating it was hypophosphorylated in the presence of ICP27 but not by the ICP27 RGG box mutants, which do not interact with SRPK1 and do not relocalize SRPK1 to the nucleus (Fig. 5D). The blot was probed with antibody to SRSF1, which showed that levels of endogenous SRSF1 were similar, so the reduced phosphorylation observed was not because of reduced expression. Thus, while WT ICP27 inhibits SRPK1 activity *in vivo*, resulting in hypophosphorylated SR proteins, RGG box mutants do not interact with SRPK1 and do not promote hypophosphorylation.

## DISCUSSION

The activity of the cellular protein SRPK1 is central to maintaining the correct level of phosphorylation of SR proteins, which in turn allows efficient splicing (3). Imbalances in posttranslational modification of SR proteins in cells harboring SRPK1 mutants not only affect splicing efficiency and alternative splicing but is also a disease phenotype in certain cancers and therefore the inhibition of SRPK1 is a drug target (1, 2). A number of viruses such as HSV-1, HBV, and HPV express proteins that bind to SRPK1 as the substrates, and some of these interacting proteins can also modulate kinase activity, which shifts host mRNA processing in favor of viral transcripts (25–27). HSV-1 ICP27 was the first viral protein identified as an SRPK1 regulator. It induces hypophosphorylation of SR proteins and redistribution of the kinase into the nucleus (27). The RGG box motif of ICP27 was identified as a requirement for SRPK1 interactions, and arginine methylation was shown to modulate the binding (51). However, it was not established whether this motif in its isolated form is sufficient for the interaction, nor was the molecular mechanism of inhibition elucidated. It was not known whether the binding occurs in a competitive manner relative to the substrate or is allosteric and whether other parts of the ICP27 protein contribute to the interaction interface. Here, we have established the molecular basis of SRPK1 interactions with ICP27 and characterized the role of the RGG box. The biophysical data indicated that the RGG box can independently interact with SRPK1, with an affinity similar to that of a native full-length substrate SRSF1 (Fig. 1). Competition between the ICP27^103–155^ peptide, which contains the RGG box and SRSF1 for binding to SRPK1 was observed directly by solution NMR (Fig. 2). The crystal structure revealed that the RGG box binds to the substrate docking groove (Fig. 3), in the same place where the SR repeats of the native substrate bind and in a configuration similar to that of a substrate (Fig. 4). The ITC experiments indicated that methylation of the RGG box at positions R138, R148, and R150 lowered affinity for SRPK1, and the structure provides an explanation for this. Within an unmodified RGG box, the side chains of R142, R144, and R148 become buried upon interaction with SRPK1; methylation at these positions is likely to introduce steric clashes, thus reducing binding affinity. Also if the RGG box interaction with SRPK1 is mediated in part by a sliding mode in order to search for an optimal residue register, in a manner analogous to the processive phosphorylation mechanism, then methylation may also interfere with transient binding events that facilitate sliding within the docking groove. Immunoprecipitation experiments analyzing SRPK1 interaction with full-length ICP27 further supported the necessity for an intact RGG box sequence for efficient interaction of the proteins and the resultant hypophosphorylation of SR proteins *in vivo* (Fig. 5). Additionally immunofluorescence data indicated that the cellular redistribution of SRPK1 by ICP27 is dependent on the RGG box, and single point mutations of arginines are sufficient to perturb SRPK1 relocalization. The inhibitory action of full-length ICP27 appeared effective in the immunoprecipitation experiments, which was also previously demonstrated via *in vitro* kinase assays (27).

In the context of infection, ICP27 appears to play a dual role in inhibiting SRPK1 action on SR protein substrates. First, the ICP27 RGG box is able to directly compete with RS domains for the docking groove of SRPK1, and we speculate that additional regions of ICP27 may further enhance affinity for SRPK1. Second, the redistribution of SRPK1 to the nucleus by ICP27 may prevent contact of the kinase with hypophosphorylated SR proteins, which are typically imported after phosphorylation into the nucleus. Therefore, through a high-affinity interaction, ICP27 is able to regulate the activity and cellular distribution of SRPK1, thus preventing the phosphorylation of SR proteins and disrupting host splicing. While global transcriptome sequencing (RNA-seq) studies have shown that ICP27 inhibits splicing of certain introns in cellular genes and also can promote the use of alternative 5′ splice sites in cellular pre-mRNAs (37), the advantages to viral infection have been less clear. Recently, it was shown through global analysis of splice junctions in cells infected with HSV-1 that there are hundreds of novel alternative splice junctions mapping to known HSV-1 spliced genes and previously unknown spliced genes, the majority of which alter the coding potential of viral genes. Splicing efficiency of the novel alternatively spliced genes revealed that splicing at the novel splice sites was efficient only in an HSV-1 ICP27 deletion mutant virus infection. In WT HSV-1-infected cells, splicing of the novel splice junctions was mainly silenced in a gene- and sequence-specific manner, suggesting that ICP27’s effects on host cell splicing ensure accuracy of the functional coding sequences of viral genes through inhibition of alternative splicing (59). Thus, the interaction of ICP27 with SRPK1 and the subsequent inhibition of splicing have important and previously unappreciated consequences on viral gene expression.

## MATERIALS AND METHODS

### Protein expression and purification.

The proteins glutathione *S*-transferase (GST)-ICP27^103–155^, GB1-SRPK1, and SRPK1 ΔNS1 were expressed in Escherichia coli. Proteins were purified by standard GST or His tag purification methods followed by size exclusion chromatography. Further details are provided in Text S1 in the supplemental material.

10.1128/mBio.02551-19.1 TEXT S1 Detailed methods for protein expression and protein purification. A detailed description of the nuclear magnetic resonance analysis and the methods for how spectra were acquired and analyzed are also provided, as are the details of the generation of the complexes of SRPK1-RGG peptide for the crystallization and how the crystals were prepared. References for the supplemental methods are also listed. Download Text S1, DOCX file, 0.1 MB. Copyright © 2019 Tunnicliffe et al. 2019 Tunnicliffe et al. This content is distributed under the terms of the Creative Commons Attribution 4.0 International license.

### Synthetic peptides.

Peptides for the ICP27 RGG box used in crystallization experiments were produced by EZBiolab (Carmel, IN, USA) comprised ICP27 residues 137 to 152 (sequence ARGGRRGRRRGRGRGG), in two forms: unmodified standard L-peptide and methylated with asymmetric dimethylation on R138, R148, and R150. Peptides used for ITC comprising ICP27 residues 103 to 155 in unmodified form and dimethylated on R138, R148, and R150 as described above were produced by Peptide Protein Research (Hampshire, United Kingdom).

### Crystallization.

To generate complexes of SRPK1 ΔNS1 with RGG box peptide ICP27^137–152^ (ICP27 residues 137 to 152) for crystallography purified 2 μM SRPK1 ΔNS1 was combined with a 6 μM peptide and incubated for 16 h at 4°C. Complex was purified using a Superdex 75 10/300 column equilibrated with buffer supplemented with 1 μM ICP27^137–152^ peptide. Protein concentrated to 260 μM grew crystals by sitting drop vapor diffusion at 4°C when mixed 1:1 with a reservoir solution of 0.09 M [NaNO_3_, 0.3 Na_2_HPO_4_, 0.3 M (NH_4_)_2_SO_4_], 0.1 M (sodium HEPES, MOPS [morpholinepropanesulfonic acid]) buffer system (pH 7.5) with 50% vol/vol GOL_P4K mix (Morpheus HT96 C7; Molecular Dimensions). Crystals were flash frozen by plunge freezing in liquid nitrogen. Further details are provided in Text S1.

### Data collection, structure determination, model building and refinement.

Data were collected from single cryo-frozen crystal SRPK1 ΔNS1-RGG box at beamline i04-1 (Diamond Light Source). Data were indexed, scaled and integrated with Xia2 (60). Phases were determined by molecular replacement using phaser with coordinates from Protein Data Bank code 1WAK (61). An automated model built against the phased map in Phenix AutoBuild was used as the basis for iterative cycles of rebuilding and refinement in COOT and Phenix.refine (62, 63), with validation with MolProbity and PDB_REDO (64, 65). ICP27 RGG box peptides were initially built as polyalanine to ensure unbiased identification of the correct register of residues. Table S3 lists data collection and refinement statistics. Cocrystallization of SRPK1 ΔNS1-RGG box was only successful with the unmodified ICP27^137–152^ peptide, which diffracted to 2.8 Å (Table S3). Coordinates were deposited in the Protein Data Bank under accession code 6FAD.

### Nuclear magnetic resonance.

Uniformly ^13^C, ^15^N-labeled ICP27^103–155^ backbone amide signal (BMRB accession no. 27341 [55]) perturbations were monitored upon additional of unlabeled GB1-SRPK1. Similarly, uniformly ^15^N-labeled SRSF1 backbone amide signals signal perturbations in ^1^H-^15^N HSQC spectra of SRSF1 were monitored upon additional of unlabeled GB1-SRPK1. For IDIS-HSQC-NMR experiments (56), a sample was prepared containing a 1:1 mixture of differentially labeled proteins, specifically 50 μM ^13^C, ^15^N-labeled ICP27^103–155^ and 50 μM ^15^N-labeled SRSF1. IDIS-HSQC spectra were acquired of this 1:1 mixture and then with unlabeled GB1-SRPK1 added to 25 μM and then to 50 μM. Data were processed in Topspin (Bruker) and analyzed in Sparky (66). Further details are in Text S1.

### Isothermal titration calorimetry.

GB1-SRPK1 and ICP27 peptides were placed into Slide-a-Lyzer G2 cassettes (Thermo Fisher) and dialyzed within identical buffer consisting of 20 mM HEPES, 150 mM NaCl, and 1 mM TCEP [Tris(2-carboxyethyl)phosphine hydrochloride] (pH 7.4). Protein concentrations were measured by absorbance at 280 nm using extinction coefficients (67) of 86,580 M^−1^ cm^−1^ and 5,500 M^−1^ cm^−1^ for GB1-SRPK1 and ICP27^103–155^, respectively. Experiments were performed at 25°C on a PEAQ-ITC (Malven instruments), using 10 μM GB1-SRPK1 in the sample cell and 120 μM ICP27 peptide in the syringe. Each experiment consisted of 19 injections: the first at 0.4 μl and the rest at 2 μl. Data were processed and fitted to a single-site binding model with Microcal PEAQ-ITC analysis (V1.00.1262).

### *In vivo* studies.

For immunoprecipitation experiments, HeLa cells grown on minimal essential medium containing 10% newborn calf serum were infected with HSV-1 wild-type strain KOS or null mutant 27-LacZ as described previously (39). Transfection of plasmid DNA was performed with Lipofectamine 2000 (Invitrogen). Cells were infected with 27-LacZ 24 h after transfection to stimulate expression of the native ICP27 promoter by the virion tegument protein VP16 as previously described (39, 68). Cells were harvested 8 h later, and immunoprecipitation was performed using GFP antibody Ab290 (Abcam) or ICP27 monoclonal antibody P1119 (Virusys) as described previously (39). Western blots were probed with anti-ICP27 antibody P1119, anti-GFP antibody JL-8 (TaKaRa Bio), or anti-β-actin antibody.

In immunofluorescence experiments, rabbit skin fibroblasts (RSF) were transfected and/or infected as indicated in the legend to Fig. 5. At 8 h after infection, cells were fixed in 3.7% formaldehyde, and immunofluorescent staining was performed as previously described (43) with ICP27 antibody P1119 or ICP4 (P1114 [Virusys]). GFP fluorescence was viewed directly. Cells were viewed with a Zeiss Axiovert S100 microscope.

For *in vivo* phosphorylation analysis, HeLa cells were transfected with GFP-SRPK1 and ICP27 RGG box mutants, as indicated in Fig. 5D, and 24 h after transfection, cells were infected with 27-LacZ for 8 h. To monitor SR protein phosphorylation, 5% of each cellular lysate was resolved by SDS-PAGE. Western blot analysis was performed with antiphosphoepitope SR antibody MABE50 (Millipore), anti-SF2 (SRSF1) antibody ab38017 (Abcam), anti-ICP27 antibody P1119, and anti-lamin B1 antibody.

### Data availability.

Cocrystallization of SRPK1 ΔNS1-RGG box coordinates have been deposited in the Protein Data Bank, under accession code 6FAD.
