# Supplementary material for: Molecular Mechanism of SR Protein Kinase 1 Inhibition by the Herpes Virus Protein ICP27
Source: mBio. 2019 Oct 22;10(5):e02551-19. doi: 10.1128/mBio.02551-19 (PMC6805999; doi:10.1128/mBio.02551-19)
Supplement: TABLE S1 [file mBio.02551-19-st001.docx]

Table S1. Thermodynamic parameters measured by isothermal titration calorimetry of the interaction between SRPK1 and non-methylated ICP27^103-155^.

| Run | [Syr]  (µM) | [Cell]  (µM) | N  (sites) | *K*_d_  (nM) | ∆H  (kcal/mol) | ∆G  (kcal/mol) | -T∆S  (kcal/mol) | Offset  (kcal/mol) | Red. Chi-Sqr.  (kcal/mol)² |
| --- | --- | --- | --- | --- | --- | --- | --- | --- | --- |
| 1 | 120 | 10 | 1.05 | 119 | -17.3 | -9.45 | 7.89 | -0.121 | 0.093 |
| 2 | 120 | 10 | 1.12 | 89.7 | -17.1 | -9.62 | 7.48 | -0.146 | 0.096 |
| 3 | 120 | 10 | 1.11 | 73.1 | -16.2 | -9.74 | 6.44 | -0.305 | 0.491 |
| Mean |  |  | 1.09 | 93.9 | -16.9 | -9.60 | 7.27 | -0.191 | 0.227 |
| SD |  |  |  | 23.2 |  |  |  |  |  |
