# Supplementary material for: Molecular Mechanism of SR Protein Kinase 1 Inhibition by the Herpes Virus Protein ICP27
Source: mBio. 2019 Oct 22;10(5):e02551-19. doi: 10.1128/mBio.02551-19 (PMC6805999; doi:10.1128/mBio.02551-19)
Supplement: TABLE S2 [file mBio.02551-19-st002.docx]

Table S2. Thermodynamic parameters measured by isothermal titration calorimetry of the interaction between SRPK1 and methylated ICP27^103-155^.

| Run | [Syr]  (µM) | [Cell]  (µM) | N  (sites) | *K*_d_  (nM) | ∆H  (kcal/mol) | ∆G  (kcal/mol) | -T∆S  (kcal/mol) | Offset  (kcal/mol) | Red. Chi-Sqr.  (kcal/mol)² |
| --- | --- | --- | --- | --- | --- | --- | --- | --- | --- |
| 1 | 120 | 10 | 0.93 | 237 | -18.9 | -9.04 | 9.87 | 0.153 | 0.121 |
| 2 | 120 | 10 | 1.00 | 272 | -18.4 | -8.96 | 9.44 | 0.182 | 0.103 |
| 3 | 120 | 10 | 1.00 | 274 | -18.0 | -8.95 | 9.07 | 0.279 | 0.098 |
| Mean |  |  | 0.98 | 261 | -18.4 | -8.98 | 9.46 | 0.205 | 0.107 |
| SD |  |  |  | 20.8 |  |  |  |  |  |
