# Supplementary material for: Molecular Mechanism of SR Protein Kinase 1 Inhibition by the Herpes Virus Protein ICP27
Source: mBio. 2019 Oct 22;10(5):e02551-19. doi: 10.1128/mBio.02551-19 (PMC6805999; doi:10.1128/mBio.02551-19)
Supplement: FIG S1 [file mBio.02551-19-sf001.docx]

Figure S1


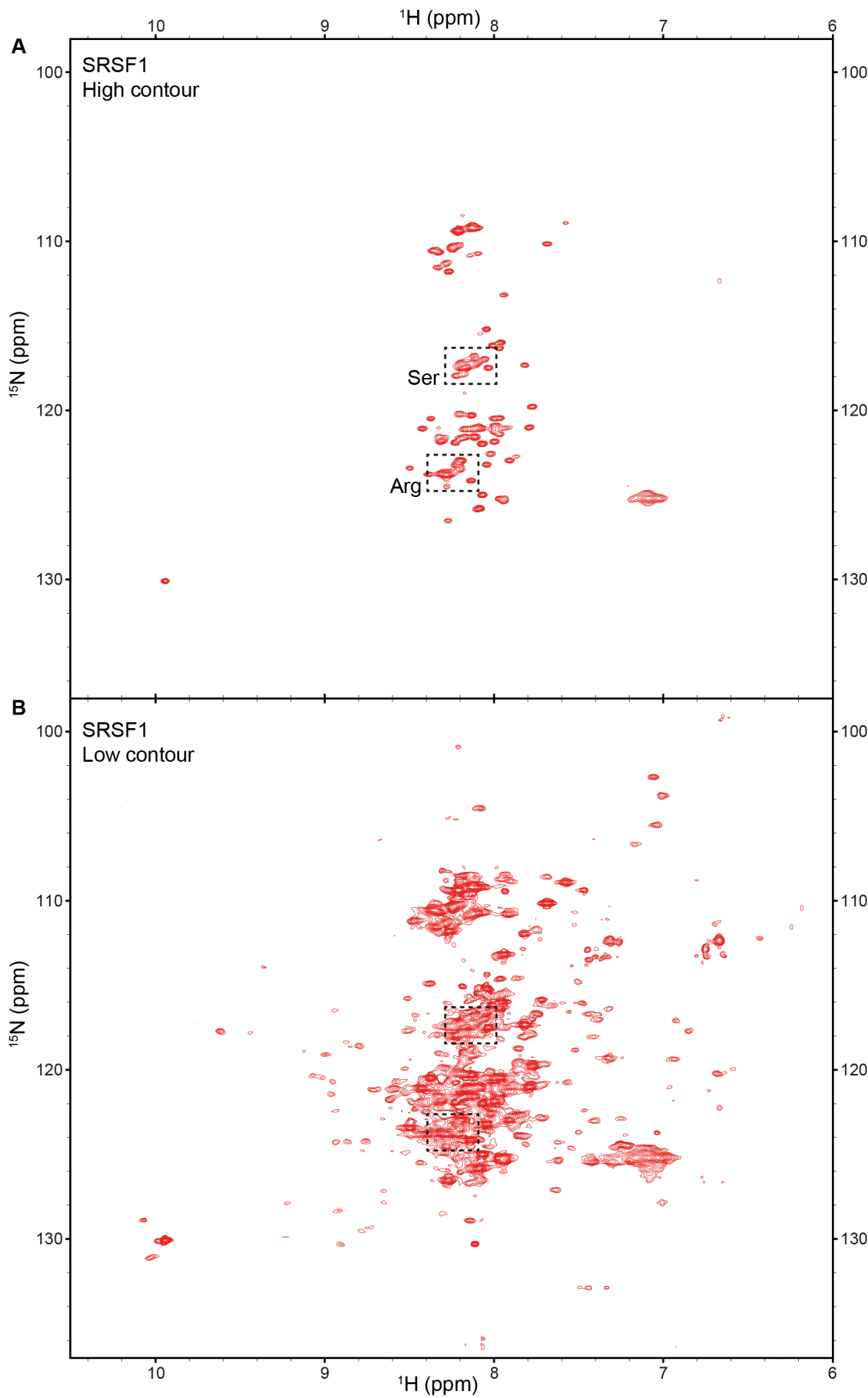


Figure S1. TROSY ^1^H-^15^N NMR correlation spectrum of uniformly [^15^N]-labelled full-length SRSF1. The same spectrum is shown with contour levels adjusted to illustrate differential peak heights. (A) High contour level only reveals sharp poorly dispersed signals from intrinsically disordered termini including the RS-repeats labelled with black dashed boxes. (B) Low contour view where broad and dispersed signals from globular RRM domains can be observed in addition to sharp signals.
