# Supplementary material for: Molecular Mechanism of SR Protein Kinase 1 Inhibition by the Herpes Virus Protein ICP27
Source: mBio. 2019 Oct 22;10(5):e02551-19. doi: 10.1128/mBio.02551-19 (PMC6805999; doi:10.1128/mBio.02551-19)
Supplement: FIG S2 [file mBio.02551-19-sf002.docx]

Figure S2


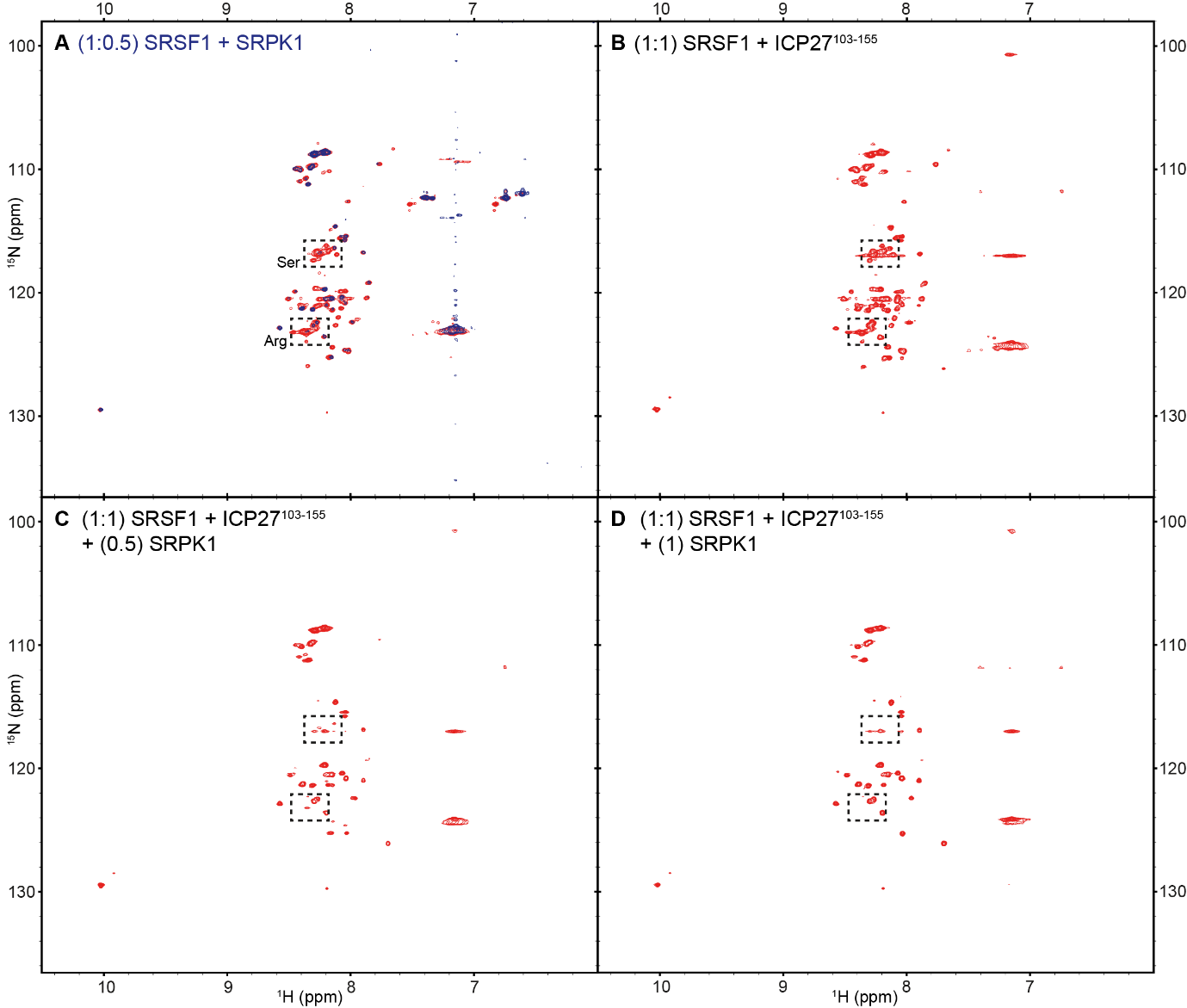
Figure S2. Interaction of uniformly [^15^N]-labeled SRSF1with unlabeled SRPK1 monitored by HSQC and IDIS-NMR ^1^H-^15^N correlation spectra. Dashed boxes mark positions of overlapped signals from the RS-repeat regions used in intensity analysis. (A) Superposition of HSQC of SRSF1 in the presence (blue) and absence (red) of SRPK1, the kinase at half the concentration of SRSF1. (B) IDIS-HSQC ^15^N-subspectra of 1:1 [^15^N]-SRSF1 and [^15^N,^13^C]-ICP27^103-155^. (C) Same sample as panel B with addition of SRPK1 to a 0.5 stoichiometric equivalent. (D) Same sample as panel C with a further addition of SRPK1 to equimolar concentration giving a ternary 1:1:1 mixture of unlabeled SRPK1, [^15^N]-SRSF1 and [^15^N,^13^C]-ICP27^103-155^_._
