# Supplementary material for: Molecular Mechanism of SR Protein Kinase 1 Inhibition by the Herpes Virus Protein ICP27
Source: mBio. 2019 Oct 22;10(5):e02551-19. doi: 10.1128/mBio.02551-19 (PMC6805999; doi:10.1128/mBio.02551-19)
Supplement: FIG S3 [file mBio.02551-19-sf003.docx]

Figure S3


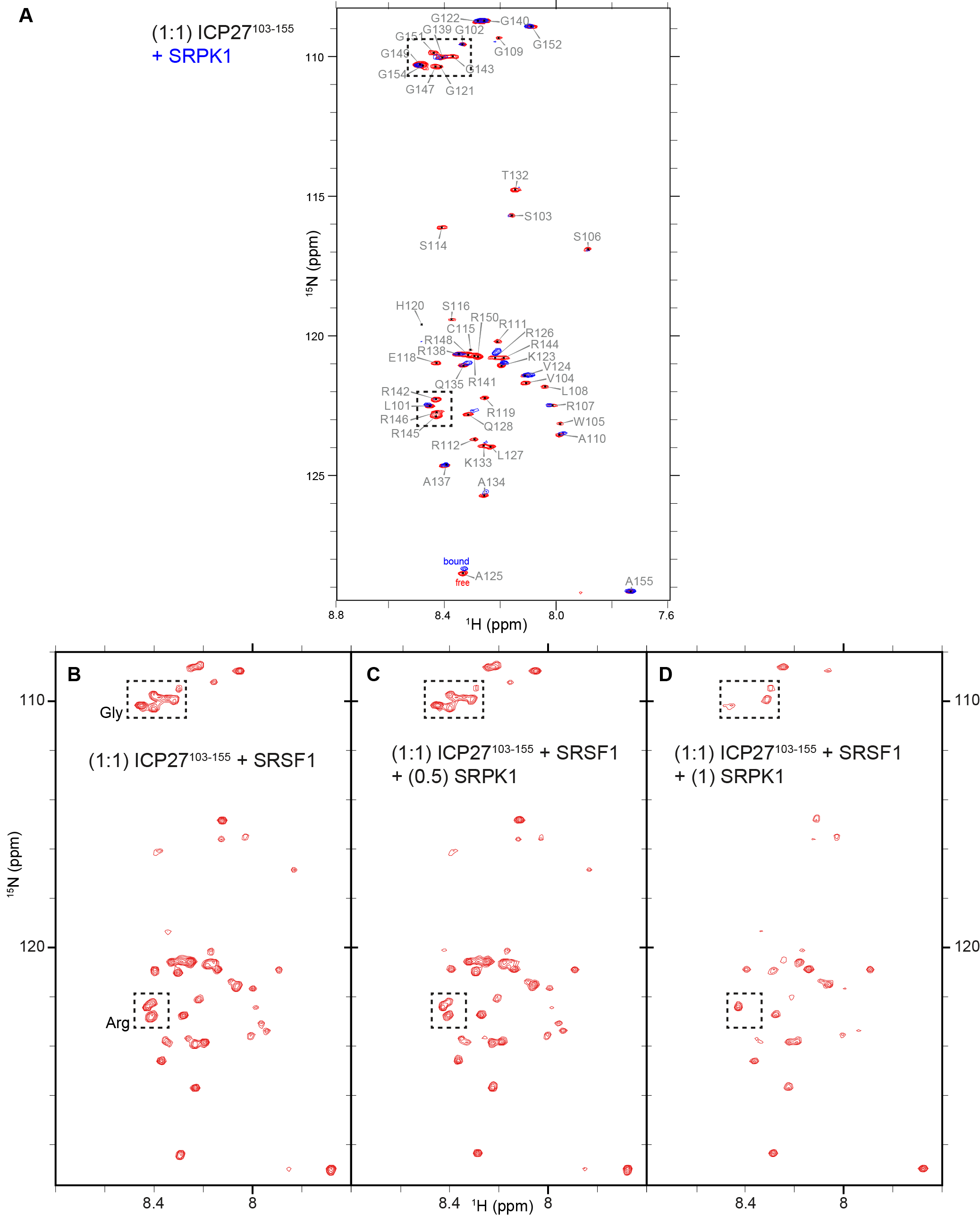


Figure S3. Interaction of uniformly [^13^C,^15^N]-labeled ICP27^103-155^ with unlabeled SRPK1 monitored by HSQC and IDIS-NMR ^1^H-^15^N correlation spectra. Dashed boxes mark positions of overlapped signals from the RGG-box signals used in intensity analysis. (A) Superposition of HSQC of ICP27^103-155^ in the presence (blue) and absence (red) of SRPK1, the kinase at half the concentration of ICP27^103-155^. (B) IDIS-HSQC ^13^C,^15^N-subspectra of 1:1 [^15^N]-SRSF1 and [^15^N,^13^C]-ICP27^103-155^. (C) Same sample as panel B with addition of SRPK1 to a 0.5 stoichiometric equivalent. (D) Same sample as panel C with a further addition of SRPK1 to equimolar concentration giving a ternary 1:1:1 mixture of unlabeled SRPK1, [^15^N]-SRSF1 and [^15^N,^13^C]-ICP27^103-155^.
