# Supplementary material for: Molecular Mechanism of SR Protein Kinase 1 Inhibition by the Herpes Virus Protein ICP27
Source: mBio. 2019 Oct 22;10(5):e02551-19. doi: 10.1128/mBio.02551-19 (PMC6805999; doi:10.1128/mBio.02551-19)
Supplement: TABLE S3 [file mBio.02551-19-st003.docx]

Table S3. X-ray crystallography data collection and refinement statistics for the SRPK1-ICP27^137-152^ structure.

|  | **SRPK1-RGG** |
| --- | --- |
| **Wavelength** | 0.9282 |
| **Resolution range** | 80.21 - 2.80 (2.90 - 2.80) |
| **Space group** | P 3_2_ 1 2 |
| **Unit cell** | 99.98 99.98 426.049 90 90 120 |
| **Total reflections** | 613946 (53315) |
| **Unique reflections** | 60676 (5984) |
| **Multiplicity** | 10.1 (8.9) |
| **Completeness (%)** | 99.95 (99.97) |
| **Mean I/sigma(I)** | 17.50 (2.54) |
| **Wilson B-factor** | 25.36 |
| **R-merge** | 0.095 (0.753) |
| **R-meas** | 0.100 (0.800) |
| **R-pim** | 0.031 (0.268) |
| **CC1/2** | 0.994 (0.812) |
| **CC*** | 0.999 (0.947) |
| **Reflections used in refinement** | 60674 (5982) |
| **Reflections used for R-free** | 2993 (303) |
| **R-work** | 0.198 (0.242) |
| **R-free** | 0.257 (0.326) |
| **CC(work)** | 0.744 (0.830) |
| **CC(free)** | 0.742 (0.706) |
| **Number of non-hydrogen atoms** | 12099 |
| **macromolecules** | 11885 |
| **ligands** | 25 |
| **solvent** | 189 |
| **Protein residues** | 1462 |
| **RMS(bonds)** | 0.008 |
| **RMS(angles)** | 0.97 |
| **Ramachandran favored (%)** | 95.34 |
| **Ramachandran allowed (%)** | 4.31 |
| **Ramachandran outliers (%)** | 0.35 |
| **Rotamer outliers (%)** | 4.83 |
| **Clashscore** | 4.78 |
| **Average B-factor** | 41.54 |
| **macromolecules** | 41.80 |
| **ligands** | 49.65 |
| **solvent** | 24.23 |
| **Number of TLS groups** | 8 |

Statistics for the highest-resolution shell are shown in parentheses.
