# Supplementary material for: Molecular Mechanism of SR Protein Kinase 1 Inhibition by the Herpes Virus Protein ICP27
Source: mBio. 2019 Oct 22;10(5):e02551-19. doi: 10.1128/mBio.02551-19 (PMC6805999; doi:10.1128/mBio.02551-19)
Supplement: FIG S4 [file mBio.02551-19-sf004.docx]

Figure S4


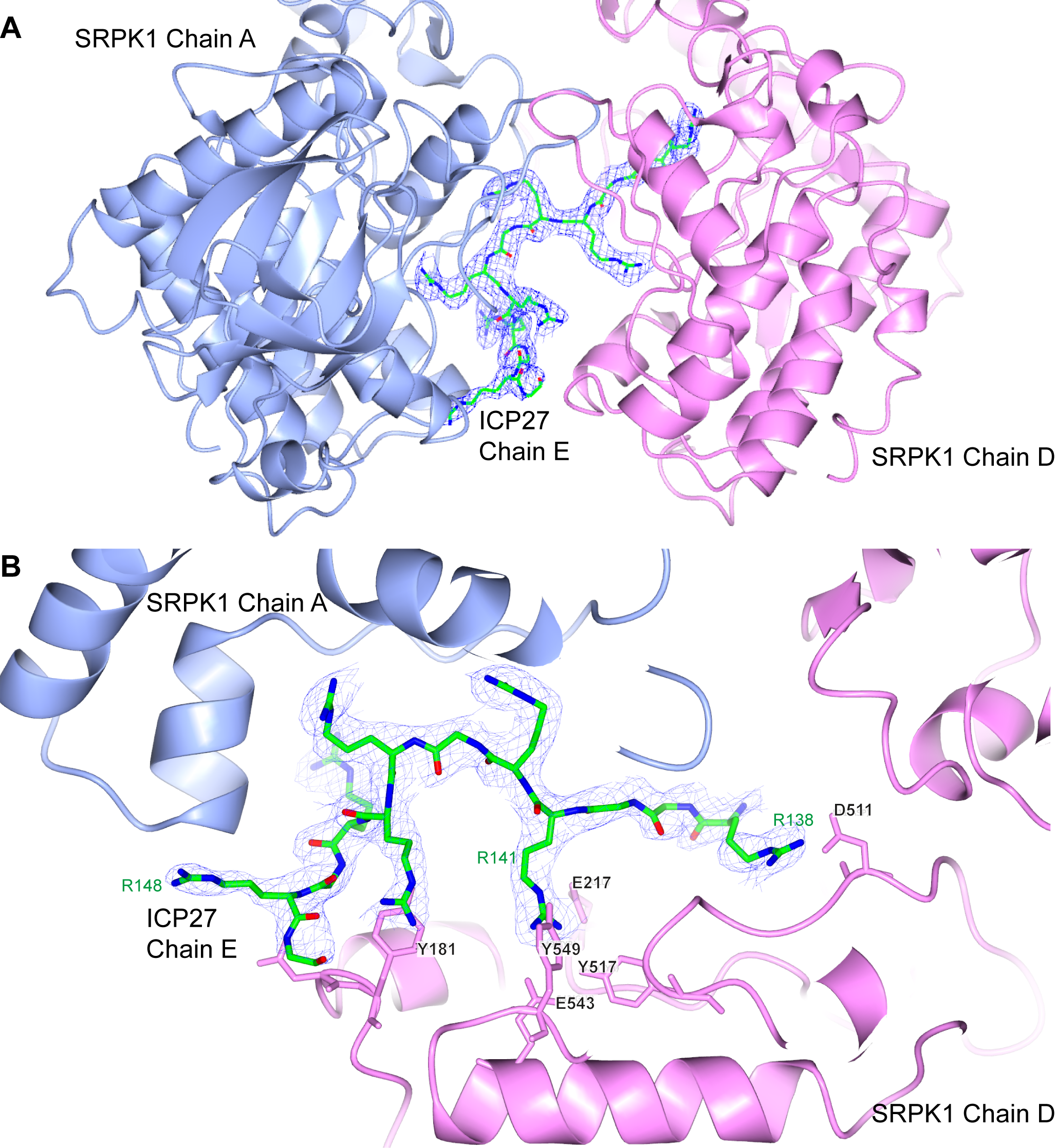


Figure S4. One ICP27 RGG-box peptide out of four present in the asymmetric unit (ASU) forms additional crystal packing contacts. Chains A (blue ribbons) and E (green sticks) are within the same ASU and chain D (pink ribbons) in an adjacent ASU. Chain E bridges the two SRPK1 molecules, with ICP27 N-terminal residues 138-141 forming apparent non-native contacts. *2F_o_-F_c_* electron density map for the ICP27 peptide is shown as blue chicken wire scaled to 1σ. (A) Overview showing the complete chains of SRPK1 chains A and D, plus ICP27 chain E. (B) Zoomed in view with residues labelled that make crystal packing contact in chains E and D.
