# Supplementary material for: Molecular Mechanism of SR Protein Kinase 1 Inhibition by the Herpes Virus Protein ICP27
Source: mBio. 2019 Oct 22;10(5):e02551-19. doi: 10.1128/mBio.02551-19 (PMC6805999; doi:10.1128/mBio.02551-19)
Supplement: TABLE S4 [file mBio.02551-19-st004.docx]

Table S4. Composition of the 4 molecular assemblies that comprise the asymmetric unit in the X-ray structure of ICP27^137-152^ in complex with SRPK1ΔNS1.

| Chain | Residues | Molecular Assembly |
| --- | --- | --- |
| A | 55-233, 479-655 | 1 |
| B | 55-233, 479-655 | 2 |
| C | 55-233, 476-655 | 3 |
| D | 55-233, 479-655 | 4 |
| E | 138-149 | 1 |
| F | 142-148 | 4 |
| G | 142-149 | 3 |
| H | 142-149 | 2 |
