# Supplementary material for: Molecular Mechanism of SR Protein Kinase 1 Inhibition by the Herpes Virus Protein ICP27
Source: mBio. 2019 Oct 22;10(5):e02551-19. doi: 10.1128/mBio.02551-19 (PMC6805999; doi:10.1128/mBio.02551-19)
Supplement: TEXT S1 [file mBio.02551-19-s0001.docx]

**Supplementary Methods**

**Protein expression.** SRSF1 synthetic DNA was obtained codon optimized for *E. coli* expression with an N-terminal Strep-tag and a C-terminal His-tag sub-cloned into pET-21a and expressed in BL21 (DE3) (Agilent Technologies). The protein constructs GST-ICP27^103-155^, GB1-SRPK1 and SRPK1ΔNS1 were expressed in *E.coli* strain BL21-CodonPlus (DE3)-RP (Agilent Technologies). Terrific Broth (Sigma) media (TB) supplemented with 50 μg/mL ampicillin or 25 μg/mL kanamycin where appropriate were inoculated with 1% v/v overnight pre-culture at 37 °C. Culture density was monitored at 600 nm until OD 0.6, then culture temperature was decreased to 20 °C and protein expression was induced with 0.25 mM IPTG and incubation continued for 16 h. Cells were pelleted by centrifugation (5000 g, 20 m). Uniformly ^13^C,^15^N or ^15^N-labeled proteins were obtained by growth in M9 minimal supplemented with ^13^C-glucose and/or ^15^N-ammonium chloride in place of TB.

**Protein purification.** GST-ICP27^103-155^ was purified with glutathione resin and cleaved from the GST fusion and cleaned up by size exclusion chromatography as detailed previously for GST-ICP27 residues 103-138 (1). Cell pellets for SRPK1 constructs were resuspended in ice cold binding buffer (50 mM Tris, 50 mM L-Arg, 50 mM L-Glu, 500 mM NaCl, pH 8.0) supplemented with 0.5% v/v Triton X-100, DNase, RNase and ethylenediaminetetraacetic acid (EDTA) free protease inhibitor (Roche). The cell suspension was lysed by sonication and clarified by centrifugation (38000 xg, 30 m, 4°C) then the supernatant was passed through a 0.2 μm filter. GB1-SRPK1 and SRPK1ΔNS1 were both purified using Ni-NTA resin, the resin was washed with binding buffer supplemented with 25 mM imidazole, protein was eluted by increasing imidazole concentration to 500 mM. Purified fractions of GB1-SRPK1 were pooled and then purified using a Superdex 200 26/600 column equilibrated in GF buffer (20 mM HEPES, 50 mM L-Arg, 50 mM L-Glu, 150 mM NaCl, 1 mM TCEP, pH 7.4). For SRPK1ΔNS1 the His-tag was cleaved using thrombin agarose (Sigma) following manufacturer’s instructions and then purified on a Superdex 75 26/600 column equilibrated in GF buffer. SRSF1 cell pellets resuspended in ice cold binding buffer and lysed and centrifuged as described above for SRPK1. The pellet was then resuspended in denaturing buffer (50 mM Tris, 6 M GuHCl, 1 M NaCl, pH 8.0) and centrifuged (38000 xg, 30 m). Denatured protein was purified from the supernatant using Ni-NTA resin equilibrated in denaturing buffer, washed with the same buffer supplemented with 25 mM imidazole and then eluted with 500 mM imidazole. The protein was refolded by rapid 20-fold dilution into ice cold 100 mM MES, 500 mM L-Arg, 500 mM L-Glu, 5 mM TCEP, 1 mM EDTA, pH 6.0, centrifuged (5000 xg, 10 m, 4°C), then dialyzed exhaustively against 50 mM MES, 50 mM L-Arg, 50 mM L-Glu, 1 mM EDTA,1 mM TCEP, pH 6.0. Full length ICP27 pellet was gently resuspended on ice in binding buffer and supplemented with 1 % v/v Triton X-100, benzonaze (Sigma) and ethylenediaminetetraacetic acid free protease inhibitor (Roche). Suspension was incubated on ice with gentle mixing by pipette every 5 mins, after 30 min mixture was sonicated briefly (20%, 30 sec) on ice and centrifuged (35000 xg, 30 min, 4°C). Supernatant was passed through a 0.2 μm filter and purified on Ni-NTA resin followed by Superdex 200 size exclusion following the same method as described for GB1-SRPK1 above.

**Nuclear magnetic resonance.** Uniformly ^13^C,^15^N labeled ICP27^[103-155]^ concentrated to 0.45 mM was solubilized in 20 mM phosphate, 50 mM NaCl, 50 mM L-Arg/L-Glu/β-mercaptoethanol and 10 mM EDTA, pH 6.2. Backbone amide signal assignments from BMRB accession number 27341 (2) were used in analysis of signal perturbations in ^1^H-^15^N HSQC spectra, which were monitored upon additional of unlabeled GB1-SRPK1 dialyzed into the same pH 6.2 buffer. Uniformly ^15^N labeled SRSF1 concentrated to 250 µM was solubilized in 50 mM MES/L-Arg/L-Glu, 1 mM TCEP, 1 mM EDTA, pH 6.0 and backbone amide signals were observed in ^1^H-^15^N HSQC and TROSY spectra; to allow assignment of sharpest signals TOCSY-HSQC and NOESY-HSQC spectra were acquired with mixing times of 60 ms and 120 ms respectively. Signal perturbations in ^1^H-^15^N HSQC spectra of SRSF1 were monitored upon additional of unlabeled GB1-SRPK1 dialyzed into the same pH 6.0 buffer. For IDIS experiments (3) a sample was prepared containing a 1:1 mixture of differentially labeled proteins, specifically 50 µM ^13^C,^15^N labeled ICP27^103-155^ and 50 µM ^15^N labeled SRSF1 solubilized in 50 mM MES/L-Arg/L-Glu, 1 mM TCEP, pH 6.0. IDIS-HSQC spectra were acquired of this 1:1 mixture and then with unlabeled GB1-SRPK1 added to a final concentration of 25 µM (0.5 stoichiometry) and finally with GB1-SRPK1 added to a final concentration of 50 µM (stoichiometric). The involvement of proteins in ligand binding in IDIS experiments was assessed by analyzing the relative changes of amide signal intensities of differentially-labelled ICP27^[103-155]^ and SRSF1 upon binding of unlabeled GB1-SRPK1 using the following equation:

$$I^{rel}= \frac{I^{B}}{I^{F}}\times\frac{I_{Ref}^{F}}{I_{Ref}^{B}}$$

where $I^{B}$ and $I^{F}$ are the mean intensities of the RGG signals (from ICP27) or RS signals (from SRSF1) in the bound and free form, respectively, and $I_{Ref}^{B}$ and $I_{Ref}^{F}$ are the reference signal intensities of A125 (from ICP27) or threonine signal from T7 tag (from SRSF1). NMR spectra were recorded at 25°C on a Bruker Advance 800 MHz spectrometer equipped with a TCI cryoprobe. Data was processed in Topspin (Bruker) and analyzed in Sparky (4).

**Crystallization.** To generate complexes of SRPK1ΔNS1-RGG peptide (ICP27 residues 137-152) for crystallography, purified 2 µM SRPK1ΔNS1 was combined with a 6 µM peptide and incubated for 16 h at 4 °C. The co-complex was purified using a Superdex 75 10/300 column equilibrated in GF buffer supplemented with 1 µM RGG-peptide. Fractions were pooled and concentrated in vivaspin centrifugal devices with a 5 kDa MWCO to 260 μM and used to set up 5 × 96 crystal trials and screened by the sitting drop vapor diffusion method. A 200 nL drop of protein–peptide concentrate was mixed with 200 nL of the screen condition using a TTP Mosquito Crystal nanolitre pipetting robot. Following 7 day incubation at 4 °C the plates were manually inspected and single crystals suitable for X-ray diffraction analysis were observed in a range of conditions. SRPK1ΔNS1-RGG grew from reservoir solutions consisting of 0.09 M (NaNO_3_, 0.3 Na_2_HPO_4_, 0.3 M (NH_4_)_2_SO_4_), 0.1 M (Sodium HEPES, MOPS) buffer system, pH 7.5, 50% v/v GOL_P4K mix (Morpheus HT96 C7, Molecular Dimensions). Crystals were flash frozen by plunge freezing in liquid nitrogen prior to data collection at Diamond Light Source Ltd.

**Supplemental References**

1. Tunnicliffe RB*, et al.* (2011) Structural basis for the recognition of cellular mRNA export factor REF by herpes viral proteins HSV-1 ICP27 and HVS ORF57. *PLoS Pathog* 7(1):e1001244.

2. Tunnicliffe RB, Tian X, Storer J, Sandri-Goldin RM, & Golovanov AP (2018) Overlapping motifs on the herpes viral proteins ICP27 and ORF57 mediate interactions with the mRNA export adaptors ALYREF and UIF. *Sci Rep* 8(1):15005.

3. Golovanov AP, Blankley RT, Avis JM, & Bermel W (2007) Isotopically discriminated NMR spectroscopy: a tool for investigating complex protein interactions in vitro. *J Am Chem Soc* 129(20):6528-6535.

4. Lee W, Tonelli M, & Markley JL (2015) NMRFAM-SPARKY: enhanced software for biomolecular NMR spectroscopy. *Bioinformatics* 31(8):1325-1327.
